# Prevalence and risk factors for faecal carriage of multidrug resistant *Escherichia coli* among slaughterhouse workers

**DOI:** 10.1038/s41598-021-92819-3

**Published:** 2021-06-25

**Authors:** Mabel Kamweli Aworh, Oluwadamilola Abiodun-Adewusi, Nwando Mba, Birgitte Helwigh, Rene S. Hendriksen

**Affiliations:** 1 Department of Veterinary and Pest Control Services, Federal Ministry of Agriculture and Rural Development, Abuja, Nigeria; 2 Nigeria Field Epidemiology and Laboratory Training Programme, Abuja, Nigeria; 3 National Agency for Food and Drug Administration and Control (NAFDAC), Lagos, Nigeria; 4 National Reference Laboratory, Nigeria Center for Disease Control, Abuja, Nigeria; 5 Department for Global Surveillance, National Food Institute, Technical University of Denmark, Kgs. Lyngby, Denmark; 6 WHO, FAO, EU, Reference Laboratory for Antimicrobial Resistance, National Food Institute, Technical University of Denmark, Kgs. Lyngby, Denmark

**Keywords:** Microbiology, Risk factors

## Abstract

The increasing occurrence of antimicrobial-resistant *Escherichia coli* in human and animal population has become a global public health problem that requires immediate intervention. We aimed to investigate prevalence and risk factors for faecal carriage of drug-resistant *E. coli* among slaughterhouse workers. We conducted this cross-sectional study among 118 apparently healthy workers in the largest slaughterhouses in Abuja and Lagos from July to December 2020. *E. coli* was isolated from stool samples of slaughterhouse workers and antimicrobial susceptibility testing performed using the Kirby-Bauer disk diffusion method. Multi-drug resistance (MDR) was defined as resistance to three or more classes of antibiotics. Majority were males: 88.1% (n = 104), aged > 41 years: 28.8% (n = 34), married: 70.3% (n = 83), and were butchers: 53.4% (n = 63). Prevalence of MDR *E. coli* was 50% (n = 59), highest among butchers compared to slaughterhouse cleaners. Of 75 *E. coli* isolates identified, 25.3% (n = 19) were ESBL producers; 78.7% (n = 59) were MDR. Keeping animals (p = 0.01); eating at the slaughterhouse (p = 0.03) and collecting waste (p = 0.02) remained independent risk factors for acquiring MDR *E. coli*. Prevalence of resistant *E. coli* was highest among butchers and associated with keeping animals at home, eating at work, and waste-collection. Hand-hygiene and responsible use of antibiotics among slaughterhouse workers should be encouraged.

## Introduction

Globally, *Escherichia coli,* which is known as an indicator bacteria, is a common source of foodborne illnesses especially in sub-Saharan Africa including Nigeria^1,2^. The emergence of multi-drug resistant (MDR) foodborne pathogens has further worsened the public health implications of antimicrobial resistance (AMR) resulting in increased length of hospital stay, mortality, and economic burden^3^. Sub-Saharan Africa, particularly Nigeria is not exempted from the challenges posed by AMR^1,2^ as a review conducted in West Africa reported a majority of the studies were from Nigeria^4^. The World Health Organization (WHO) has reported that a high level of AMR observed in the human population is due to misuse or abuse of antimicrobials in the food and agriculture sectors^5^.

Food, particularly meat is a major source of MDR-*E. coli* among humans^6,7^. In many developing economies, food-producing animals are slaughtered and processed under very poor hygienic conditions^8^, hence, constituting an occupational risk to slaughterhouse workers (SHWs). MDR-*E. coli*, often detected in the guts of apparently healthy individuals, may not directly cause disease, but serve as a reservoir of AMR genes^9,10^. These resistant genes can further spread to other Gram-negative pathogenic bacteria in the gut and potentially become harmful hence causing disease but can also spread due to open defecation and poor hygiene^11^.

Slaughterhouses in some settings are hotspots for MDR pathogens, facilitating their spread to the environment^12^. Evidence shows that SHWs are at risk of being exposed to certain bacterial infections because of their occupation^13,14^. Antimicrobial drugs (AMDs) are also easily accessible to livestock farmers without any prescription from the veterinarian, thus, encouraging antimicrobial use (AMU) indiscriminately in food-producing animals in Nigeria due to easy access^15^.

Few studies conducted in Nigeria have examined the presence of MDR-*E.coli* in human patients and food-producing animals, however, the risk factors for faecal carriage of MDR-*E. coli* among SHWs is not yet known^16,17^. A recent review has reported that human exposure to environmental sources was an important risk factor for the acquisition of MDR-*E. coli*^10^. Most slaughterhouse environments in Nigeria are poorly managed with very poor sanitary conditions, hence promoting the spread of resistant pathogens^18^. Nigeria has a growing population of livestock characterized by an extensive system of management making it difficult to track AMU in this food-animal population because of their constant movement in search of food. Besides, there are problems of illegal importation and sale of antimicrobials for use in livestock^19^. It is also practically impossible to estimate the public health risks encountered by SHWs when they share the same environment with livestock and their waste products^2^.

There is increased AMU among livestock in Nigeria with a resultant increase in the development of AMR among enteric pathogens and indicator bacteria such as *E. coli*. It is therefore imperative to generate data on the resistance profile of indicator bacteria among apparently healthy individuals exposed to livestock by their occupation for proper diagnosis and treatment of bacterial infections.

We hypothesized that livestock harboring MDR-*E. coli* facilitates the transmission of the pathogen to workers exposed to livestock and the slaughterhouse environment. This study aimed to investigate the prevalence and risk factors for faecal carriage of MDR-*E. coli* among SHWs to generate baseline data for the establishment of the AMR Animal-Health Surveillance system in Nigeria. Prevalence was determined by antimicrobial susceptibility testing while the risk was accessed based on interviews and multivariate analysis.

## Results

Of the 118 SHWs interviewed, majority were males: 88.1% (n = 104), aged > 41 years: 28.8% (n = 34), married: 70.3% (n = 83), Muslims: 70.3% (n = 83), had Islamic education: 31.4% (n = 37), lived in urban areas: 96.6% (n = 114), of Hausa ethnicity: 73.7% (n = 87) and were butchers: 53.4% (n = 63) (Table 1).

**Table 1:** Demographic characteristics of slaughterhouse workers in Abuja and Lagos—Nigeria, 2020.

Variables	n	%
**Age group (years)**		
18–25	23	19.5
26–33	29	24.6
34–41	32	27.1
> 41	34	28.8
**Marital status**		
Divorced	1	0.9
Married	66	54.6
Single	55	45.5
Widowed	2	1.7
**Educational level**		
None	6	5.1
Islamic education	37	31.4
Primary	17	14.4
Secondary	34	28.8
Tertiary	24	20.3
**Occupation**		
Butchers	63	53.4
Slaughterhouse cleaners	21	17.8
Vets/para-vets	11	9.3
Meat carriers	10	8.5
Meat sellers	6	5.1
Livestock traders	5	4.2
Security	2	1.7
**Duration of work**		
0–5 years	35	29.7
≥ 6 years	83	70.3
**Location**		
Abuja	84	71.2
Lagos	34	28.8
**Residence**		
Rural	4	3.4
Urban	114	96.6
**Religion**		
Christianity	35	29.7
Islam	83	70.3
**Ethnicity**		
Ibo	4	3.4
Hausa	87	73.7
Yoruba	18	15.3
Others	9	7.6

### Prevalence of MDR *E. coli* among slaughterhouse workers

Fifty-nine SHWs of the 118 included in the study tested positive for MDR-*E. coli* giving a prevalence of 50%. A majority of these, 69.5% (n = 41) resided in Abuja and the highest prevalence of 50.9% was among butchers (prevalence OR = 1.00, 95% confidence interval [CI] = 0.37–2.69; p = 0.00) when compared to slaughterhouse cleaners.

**Figure 1:**
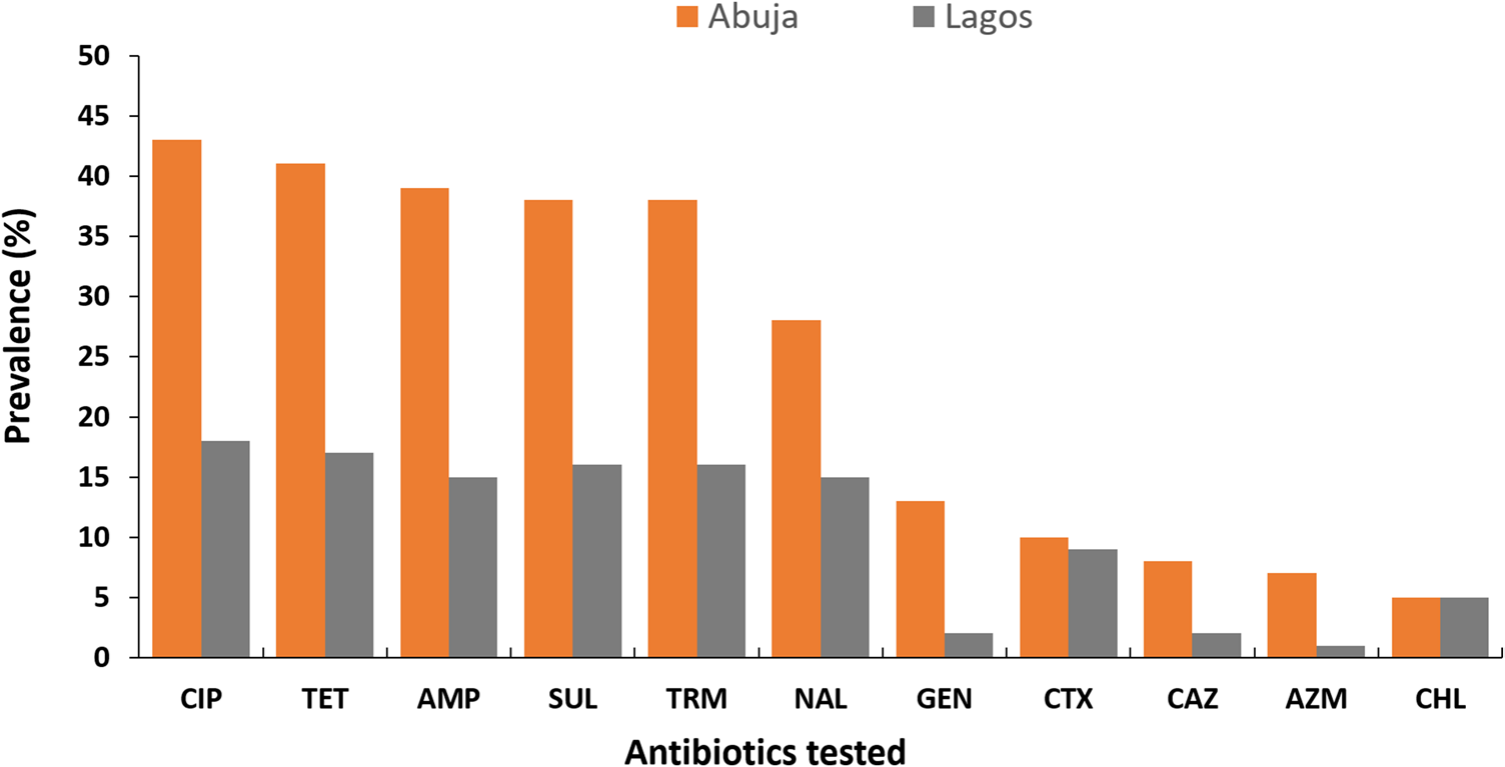
Prevalence of MDR *E. coli* isolated from stool samples of slaughterhouse workers in Abuja and Lagos slaughterhouses, 2020. Bars represent the proportion of samples containing at least one MDR *E. coli* isolate with 95% confidence intervals. Data were obtained from two sources: Abuja and Lagos slaughterhouses. *AMP* ampicillin, *AZM* azithromycin, *CAZ* ceftazidime, *CTX* cefotaxime, *CHL* chloramphenicol, *CIP* ciprofloxacin, *GEN* gentamicin, *NAL* nalidixic acid, *SUL* sulfonamides, *TET* tetracycline, and *TRM* trimethoprim.

Out of 75 *E. coli* isolates identified, 25.3% (n = 19) were ESBL-*E. coli* while 78.7% (n = 59) were MDR. The isolates were resistant to ciprofloxacin: (81.3%, n = 61), tetracycline: (77.3%, n = 58), sulfonamides: (72%, n = 54), trimethoprim: (72%, n = 54), ampicillin: (72%, n = 54), nalidixic acid: (57.3%, n = 43), cefotaxime: (25.3%, n = 19), gentamicin: (20%, n = 15), chloramphenicol: (13.3%, n = 10), ceftazidime: (13.3%, n = 10), and azithromycin: (10.7%, n = 8) (Fig. 1).

All the *E. coli* isolates were 100% susceptible to cefoxitin, colistin, imipenem, meropenem, and nitrofurantoin. The maximum number of ESBL-*E. coli* was isolated from butchers: 57.9% (n = 11) when compared to other SHWs. The resistance rates of the *E. coli* isolates to ciprofloxacin, ampicillin, tetracycline, sulfonamides, trimethoprim, nalidixic acid, cefotaxime, and chloramphenicol were higher among butchers when compared to the rest of the SHWs. All the isolates were, however, sensitive to cefoxitin, colistin, imipenem, meropenem, and nitrofurantoin (Table 2).

**Table 2:** Resistance profile of *E. coli* isolates recovered from slaughterhouse workers at Abuja and Lagos slaughterhouses to the antibiotics tested.

Drug class	Drug	Resistance breakpoints, mm	Overall, n = 75 (%)	Butchers, n = 42 (%)	Others, n = 33 (%)
Quinolones	Ciprofloxacin (5 μg)	≤ 20	61 (81.3)	32 (76.2)	29 (87.9)
	Nalidixic acid (30 μg)	≤ 13	43 (57.3)	22 (52.4)	21 (63.6)
Tetracyclines	Tetracycline (30 μg)	≤ 11	58 (77.3)	30 (71.4)	28 (84.9)
Folate pathway antagonists	Sulfonamides (300 μg)	≤ 12	54 (72.0)	28 (66.7)	26 (78.8)
	Trimethoprim (5 μg)	≤ 10	54 (72.0)	28 (66.7)	26 (78.8)
Penicillins	Ampicillin (10 μg)	≤ 13	54 (72.0)	32 (76.2)	22 (66.7)
Cephalosporins	Cefotaxime (30 μg)	≤ 22	19 (25.3)	11 (26.2)	8 (24.2)
	Ceftazidime (30 μg)	≤ 17	10 (13.3)	5 (11.9)	5 (15.2)
Aminoglycosides	Gentamicin (10 μg)	≤ 12	15 (20.0)	5 (11.9)	10 (30.3)
Phenicols	Chloramphenicol (30 μg)	≤ 12	10 (13.3)	6 (14.3)	4 (12.1)
Macrolides	Azithromycin (15 μg)	≤ 12	8 (10.7)	3 (7.2)	5 (15.2)
Resistance to 3 or more classes of antibiotics	MDR	n/a	59 (78.7)	30 (71.4)	29 (87.9)

**Figure 2:**
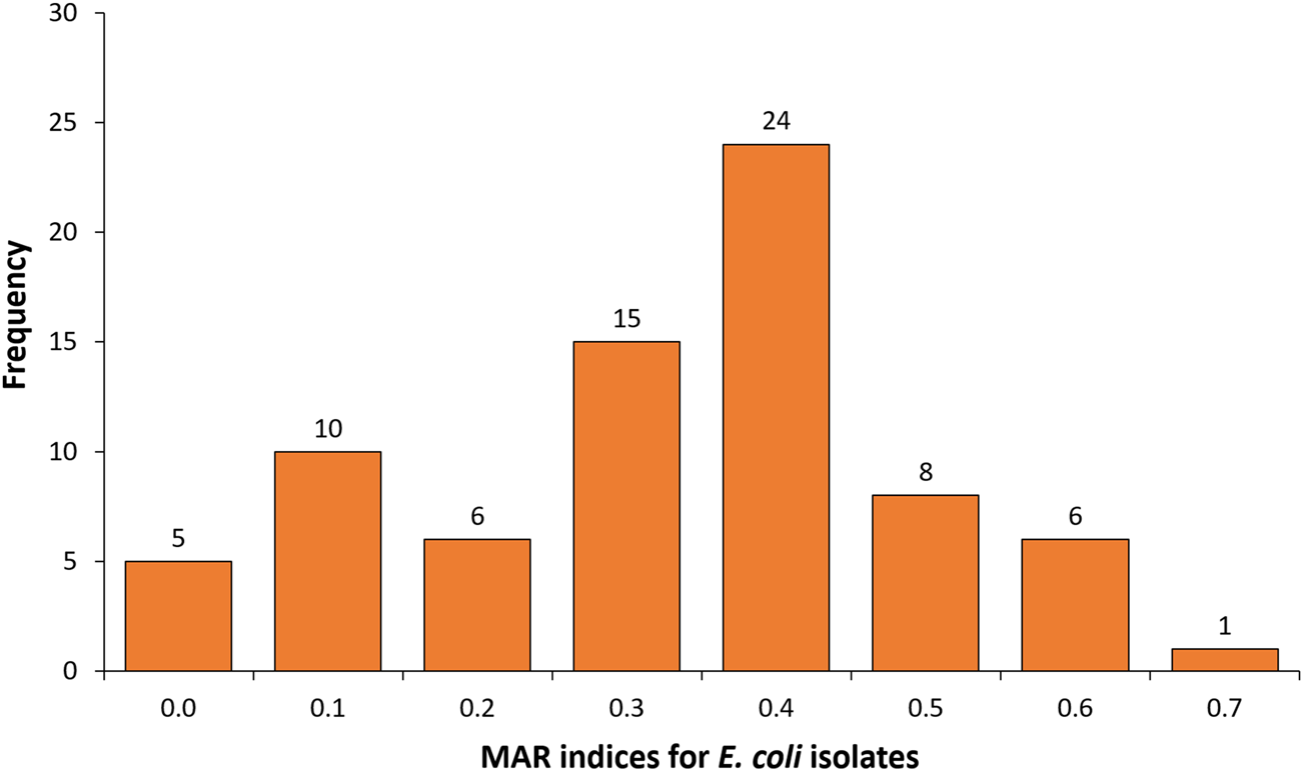
Multiple antibiotics resistance (MAR) indices for *E. coli* recovered from Slaughterhouse workers in Abuja and Lagos-Nigeria, 2020. This bar chart demonstrates the increase in MAR indices observed for the *E. coli* isolates. Each bar represents the frequency recorded for the different MAR indices. The highest frequency (n = 24) was recorded against a MAR index of 0.4.

In this study, 6.6% (n = 5) of the isolates were susceptible to all the AMDs tested, 4% (n = 3) were resistant to only one AMD while 9.3% (n = 7) were resistant to only two AMDs. Surprisingly one isolate was resistant to 11 different AMDs including those of critical importance for human health. In 72% (n = 54) of the isolates, a MAR index greater than 0.2 was observed (Fig. 2).

Among the 19 ESBL-*E. coli* isolates, high resistance was observed for cefotaxime: 100% (n = 19), ciprofloxacin: 94.7% (n = 18), tetracycline: 94.7% (n = 18), sulfonamides: 94.7% (n = 18), trimethoprim: 94.7% (n = 18), ampicillin: 94.7% (n = 18) and nalidixic acid: 78.9% (n = 15).

### Antimicrobial use patterns of slaughterhouse workers

Among 59 SHWs colonized with MDR-*E. coli*, 61% (n = 36) reported that they had taken antibiotics in the last month before stool collection. Less than half 39% (n = 23) reported that the last antibiotics they had taken were Ampiclox (a combination of ampicillin and cloxacillin) especially when they had a knife cut. About a third 35.6% (n = 21) of the workers reported that they self-prescribed antimicrobials more than three times in the last three months before sampling. A majority 93.2% (n = 55) reported that they usually purchased antibiotics from a chemist although 89.8% (n = 53) reported that they usually received direction for use from the pharmacist. More than two-thirds 71.2% (n = 42) reported that they usually completed their dosage while 30.5% (n = 18) reported that they stopped their medication whenever they felt better.

### Risk factors for MDR-*E. coli* among SHWs

Occupational hazards reported among the SHWs were quite a few. Nearly half (49.2%; n = 58) of the workers reported that a major hazard they faced was knife cuts while slaughtering the animals. Over half (56.9%; n = 33) reported that they however had to continue processing slaughtered animals even after sustaining a knife cut, hence their wounds get exposed to infection. Nearly half (48.3%; n = 57) reported that they often handle fetuses recovered from slaughtered pregnant animals.

The factors associated with MDR-*E. coli* among SHWs were: aged over 41 years old (OR 2.79, 95% CI 1.21–6.45); keeping animals at home (OR 2.13, CI 1.02–4.44); eating at the slaughterhouse while working (OR 2.13, CI 1.02–4.44); collecting slaughterhouse waste (OR 6.53, 95% CI 1.38–30.92); and washing hands with soap (OR 0.42, 95% CI 0.19–0.93) (Table 3).

**Table 3:** Bivariate analysis of factors associated with multidrug-resistant *E. coli* recovered from slaughterhouse workers in Abuja and Lagos-Nigeria, 2020.

Work exposure	MDR (Yes)	MDR (No)	Odds ratio (95% confidence level)	p value
**Age**				
≥ 41 years	23	11	2.79 (1.21–6.45)	0.02*
18–41 years	36	48		
**Level of education**				
Less than secondary school	29	31	0.87 (0.42–1.80)	0.71
Secondary and above	30	28		
**Occupation**				
Butcher	30	33	0.82 (0.40–1.68)	0.58
Others	29	26		
**Work exposure (years)**				
≥ 6	41	42	0.92 (0.42–2.03)	0.84
1–5	18	17		
**Slaughtering animal after sustaining an injury**				
Yes	18	15	1.80 (0.63–5.16)	0.27
No	10	15		
**Keeping animals at home**				
Yes	34	23	2.13 (1.02–4.44)	0.04*
No	25	36		
**Eating while working at slaughterhouse**				
Yes	36	25	2.13 (1.02–4.44)	0.04*
No	23	34		
**Collecting slaughterhouse waste**				
Yes	11	2	6.53 (1.38–30.92)	0.01*
No	48	57		
**Washing hands with soap**				
Yes	19	34	0.42 (0.19–0.93)	0.03*
No	28	21		
**Diarrhoea in last 3 months**				
Yes	23	27	0.76 (0.36–1.57)	0.46
No	36	32		

^*^Values that were significant at bivariate analysis.

Five factors that were statistically significant at bivariate analysis (p ≤ 0.05) were included in the unconditional logistic regression model used in this study. These factors include age over 41 years, keeping animals at home, eating at the slaughterhouse while working, collecting slaughterhouse waste, and washing hands with soap. There were fewer female workers hence gender was considered as a potential confounder and included in the final model used for analysis. Using a stepwise elimination approach and controlling for gender, all factors remained statistically significant in the final logistic regression model. We identified four independent risk factors and one protective factor. Being ≥ 41 years (adjusted OR [AOR] = 4.47, 95% CI  1.59–12.60); keeping animals at home; (AOR = 4.04, 95% CI  1.48–11.05); eating at the slaughterhouse (AOR = 3.01, 95% CI  1.14–7.91) and collecting slaughterhouse waste (AOR = 10.31, 95% CI  1.54–69.10) remained independent risk factors for faecal carriage of MDR-*E. coli* among SHWs. Washing hands with soap (AOR = 2.72, 95% CI  1.06–6.96), however, remained a protective factor against MDR-*E. coli* among SHWs (Table 4).

**Table 4:** Factors found to be significantly associated with MDR *E. coli* among slaughterhouse workers in Abuja and Lagos, Nigeria 2020 in a logistic regression model.

Work exposure factors	Adjusted odds ratio	95% confidence interval	p value
Age > 41 years	4.47	1.59–12.60	0.01*
Keeping animals at home	4.04	1.48–11.05	0.01*
Eating at the slaughterhouse while working	3.01	1.14–7.91	0.03*
Collecting slaughterhouse waste	10.31	1.54–69.10	0.02*
Washing hands with soap	2.72	1.06–6.96	0.04^#^

*Independent risk factors for MDR *E. coli* that remained significant in the logistic regression model. ^#^Only protective factor against MDR *E. coli* that was significant in the final logistic regression model used for the multivariate analysis. Gender was included in the final model to control for possible confounders.

Unconditional logistic regression analysis showed that SHWs had 4 times the odds of shedding MDR-*E. coli* in their stool if they were ≥ 41 years of age (p = 0.01), kept animals at home (p = 0.01), and 3 times more likely if they ate at the slaughterhouse while processing slaughtered cattle (p = 0.03). Workers were 10 times more likely to shed MDR-*E. coli* in their stool if they collected slaughterhouse waste (p = 0.02). They were 3 times more likely to be protected from being colonized by MDR-*E. coli* if they washed their hands with soap and clean water (p = 0.04).

## Discussion

To the best of our knowledge, this is the first study to document the prevalence and risk factors for faecal carriage of MDR-*E. coli* among SHWs in Abuja and Lagos, Nigeria. The present study recorded a high prevalence of 50% for MDR-*E. coli* among SHWs in both locations when compared with 36.6% and 36.8% observed in the general population respectively^23,24^. A similar study conducted among poultry workers in Abuja reported a lower prevalence of 39.7% for MDR-*E. coli*^9^*.* The high prevalence observed in the present study might be due to AMU in animals which have been associated with the development of bacterial resistance in humans exposed to these food-producing animals^25–27^. The present study demonstrates that 72% of MDR-*E. coli* recovered from the SHWs had a MAR index above 0.2. This implies that the isolates may have been recovered from sources with a high frequency of AMU^28^ although multiple AMR is usually detected when plasmids encode AMR phenotype^22^.

Our findings show a high prevalence of MDR-*E. coli* but a low prevalence of ESBL-*E. coli* colonization in the study population possibly because ceftazidime and cefotaxime are not some of the commonly self-prescribed antimicrobials in the present study. This supports the findings from a survey in the Mekong Delta in Vietnam where a prevalence of 81.3% was observed for MDR-*E. coli*, while a prevalence of 3.2% was recorded for ESBL-*E. coli*^29^. In contrast to our findings, a study conducted in Pakistan reported a much higher ESBL-*E. coli* prevalence among cattle and healthy individuals^30^. A related study conducted in southern Nigeria also reported a much higher ESBL and MDR-*E. coli* prevalence among pig SHWs^31^.

This study observed that the MDR-*E. coli* isolates recovered from SHWs were resistant to ciprofloxacin, tetracycline, ampicillin, sulfonamides, trimethoprim, and nalidixic acid. Inappropriate AMU in humans and animals has been documented in the literature to promote a selective increase in some bacterial populations as well as dissemination of resistant strains^32,33^. A higher ciprofloxacin resistance compared to nalidixic acid resistance was observed among MDR-*E. coli* isolates supporting recent reports of a similar study in southern Nigeria where a high frequency of ciprofloxacin resistance was observed in isolates recovered from pig slaughterhouse workers^31^. A possible explanation for this observation is likely due to *qnr* genes where *qnr*S1 is the most common in Africa especially since fluoroquinolone resistance in different regions have different characteristics^34^. This is not surprising as ciprofloxacin is one of the most commonly prescribed quinolones that is affordable and available over the counter in Nigeria^35^. The MDR-*E. coli* isolates were susceptible to meropenem and imipenem consistent with recent studies conducted among pig slaughterhouse workers in southern Nigeria^31^. This is not surprising as these antimicrobials are normally given as intravenous injections however, resistance started to emerge in poultry and pigs in the EU with VIM and IMP genes detected in *E. coli* and Salmonella including a few Oxa-48 genes^36^. Acquired MDR does not occur only in pathogenic bacteria but in the microflora of exposed individuals and animals. According to WHO, the important drivers for the colonization of MDR pathogens is overuse and misuse of antimicrobial drugs^5^ although studies have reported that occupation, diet, health, sanitation, and cultural traditions are also drivers for AMR^37^. This could probably explain the high prevalence of MDR-*E. coli* observed in the present study especially since some SHWs reported self-prescription of antibiotics, incomplete dosages, poor hygiene, and sanitation amongst others.

A related study among pig SHWs in Europe reported that the workers were more likely to be colonized by resistant bacteria if they were involved in the early slaughtering processes^38^. This supports our study results as butchers who are involved in these early processes were the most affected possibly because of close contact with gut content at the beginning of slaughtering especially on open slabs.

Factors associated with MDR-*E. coli* in the present study were age, exposure to animals at home, eating at the slaughterhouse while working, collecting waste, and hand hygiene. While the former are independent risk factors, the latter is a protective factor. A related study done in Nigeria among poultry workers reported that a history of diarrheoa and work exposure of over 10 years were risk factors for feacal carriage of MDR-*E. coli*^9^ however the present study did not observe any association with these factors among SHWs. A similar study done among poultry SHWs confirms that some risks do exist from occupational exposure for the acquisition of MDR-*E. coli*^39^.

Washing hands with soap was found to be a protective factor against faecal carriage of MDR-*E. coli* among the workers. According to WHO, lack of clean water and sanitation facilitates the spread of antimicrobial-resistant microbes^5^. It has been documented that washing hands with soap and clean water is very effective in removing faecal bacteria from the hands^40^ confirming our study results. The importance of hand hygiene and sanitation should be encouraged in the fight against AMR.

The mechanism of AMR spread from animals to humans is not clear however, literature shows that animals are reservoirs for *E. coli* found in humans^41^. Our results show that SHWs were colonized by MDR-*E. coli* resistant to commonly used antibiotics in humans and livestock. In addition, we identified slaughterhouse waste collection as a risk factor for the acquisition of MDR-*E. coli* among SHWs. Poor sanitary slaughterhouse environment^31,42^ and occupational exposure to food animals may have been responsible for this observation. There is a paucity of studies on risk factors for MDR-*E. coli* in the target population. Some studies reported travel history and diet as risk factors for faecal carriage of MDR-*E. coli* however the present study did not consider these factors^10,43^.

There are potential limitations in this study that could be addressed in future research. First, the study focused on the phenotypic characterization of the *E. coli* isolates to determine the resistance profile of the slaughterhouse workers. Secondly, the study determined work exposure factors as well as the antimicrobial use history of the slaughterhouse workers that put them at risk of being colonized with MDR *E. coli.* Future studies could explore the possible origin of MDR observed among the slaughterhouse workers as well as the genomic characterization of the isolates to identify the genes, determine clonality and perform cluster analysis for traceability.

Apparently healthy SHWs could harbor and shed MDR *E. coli* in their stool hence posing as a public health hazard to the general population. Prevalence of resistant *E. coli* was highest among butchers. Risk factors for faecal carriage of MDR *E. coli* were age, animal exposure at home, eating at work, and waste collection. Handwashing with soap and clean water was protective against acquiring MDR *E. coli* among the workers. This study provides additional evidence demonstrating the occupational hazards for people working in slaughterhouses. We recommend that competent authorities organize regular AMR awareness programs targeted at SHWs to mitigate public health risks. Hand hygiene and responsible use of antibiotics among SHWs should be encouraged.

## Methods

### Study area

This study was conducted in two major cities; Abuja metropolis, North-Central part of Nigeria (9.01145° N, 7.57961° E) with a population of approximately 3 M people and Lagos city, South-Western Nigeria (6.64983° N, 3.32023° E) inhabited by 14 M people, respectively.

### Study design

A cross-sectional study approach was applied among 118 apparently healthy workers who were randomly selected from the two largest slaughterhouses situated in Abuja (84 samples) and Lagos (34 samples) from July to December 2020. All SHWs aged ≥ 18 years, who volunteered were enrolled as study participants. All study participants signed the informed consent form explaining the purpose and benefits of the study before sample collection. An open data kit (ODK) installed on a smartphone was used to administer a structured interviewer-administered questionnaire to SHWs for the data collection on demographic characteristics and exposure factors.

### Sampling method

The largest slaughterhouses each in Abuja and Lagos were selected based on the daily volume of livestock slaughter. Using a simple random sampling method, we selected workers from both slaughterhouses for the study. We collected freshly passed stool samples (one sample per worker) using sterile stool containers and transported the samples in cool boxes to the National Reference Laboratory (NRL), Nigeria Centre for Disease Control, Abuja. The samples collected in Abuja were processed within 3 h of sample arrival in the laboratory for the presence of *E. coli.* Samples collected in Lagos were stored at − 80 °C thereafter they were transported to the NRL, Abuja for processing. Positivity was determined as the presence of MDR-*E. coli* in a stool sample.

### Bacteriological analysis

#### Isolation of *E. coli* isolates

Briefly, we inoculated one gram of stool sample in 9 ml of buffered peptone water (enrichment broth) and incubated at 37 °C for 24 h. Thereafter, a 10 μl loop-full culture from buffered peptone water was streaked onto the selective media, MacConkey agar, and incubated at 37 °C for 24 h. Three well-isolated suspected *E. coli* colonies were streaked, usually pink to red onto the indicative media, Eosin Methylene Blue agar, and incubated at 37 °C under aerobic conditions for 24 h^20^. Elevated, moist colonies with a greenish metallic sheen, suggestive of *E. coli,* were sub-cultured onto the non-selective media, Trypticase Soy agar plates, and incubated for 24 h at 37 °C for the isolation of pure cultures. Conventional biochemical tests were performed on all *E. coli* isolates^20^ and confirmed using a commercially available biochemical test strip, Microbact GNB 24E (Oxoid, UK), according to the Manufacturer’s instructions.

#### Antimicrobial susceptibility testing of *E. coli* isolates

The Kirby Bauer disk diffusion method was applied to perform antimicrobial susceptibility testing of the *E. coli* isolates according to the Clinical and Laboratory Standards Institute (CLSI, 2020) recommendations^21^. A panel of 16 different antibiotics of 12 classes was applied including ampicillin (10 μg), azithromycin (15 μg), tetracycline (30 μg), gentamicin (10 μg), chloramphenicol (30 μg), colistin (10 μg), sulfonamides (300 µg), trimethoprim (5 μg), cefoxitin (30 μg), ciprofloxacin (5 μg), nalidixic acid (30 μg), nitrofurantoin (300 μg), imipenem (10 μg), meropenem (10 μg), ceftazidime (30 μg), and cefotaxime (30 μg). For quality control, we used *E. coli* ATCC 25922. In this study, we defined MDR as the bacteria isolate being resistant to three or more classes of antibiotics commonly used in medical and veterinary practice in Nigeria.

#### Multiple antibiotic resistance (MAR) indexes

We calculated the MAR index for each *E. coli* isolate using the formula MAR = a/b, where *‘a’* was the number of antibiotics that the isolate was resistant to while *‘b’* was the total number of antibiotics that the isolates were subjected to^22^.

#### ESBL phenotype detection by disk diffusion

Presumptive extended-spectrum beta-lactamase-producing *E. coli* (ESBL-*E. coli*) isolates were further confirmed using the disk diffusion method according to CLSI 2020^21^ testing for synergy applying the following disks: ceftazidime (30 μg) (CAZ), ceftazidime + clavulanic acid (30/10 μg) (CAC), cefotaxime (30 μg) (CTX), cefotaxime + clavulanic acid (30/10 μg) (CEC). Following growth, the zone-diameters were measured and recorded. An increase in the zone diameter by ≥ 5 mm around the disks containing cephalosporin with the inhibitor over the disks containing solely cephalosporin indicates ESBL production (synergy). Controls: *E. coli* ATCC 25929 and *K. pneumoniae* ATCC 700603 were used as negative and positive controls, respectively.

#### Risk factor survey

We administered electronic questionnaires to SHWs using ODK collect app on a smartphone to determine the risk factors for MDR *E. coli* colonization. We assessed the association between demographics, AMU, exposure factors, and acquiring MDR-*E. coli*. We obtained permission from slaughterhouse management before administering the questionnaire to the workers. We pretested the questionnaire at the Dei-Dei slaughterhouse, however; we did not include data obtained from the pretesting in the final analysis.

### Statistical analysis

The data collected with ODK collect app was exported as a Microsoft Excel spreadsheet to a computer using the ODK briefcase tool. Data were analyzed using Epi Info version-7 by computing frequencies and proportions. To identify risk factors for MDR-*E. coli*, we performed bivariate analysis using odds-ratios (OR) as a measure of association. Risk factors identified as statistically significant at bivariate analysis were included in an unconditional logistic regression model used for multivariate analysis at a 5% significance level. All relevant data are within the paper and available as supporting information (Supplementary Information 1).

### Ethics approval

We obtained ethical approval from the Federal Capital Territory (FCT) Health Research Ethics Committee (Approval Number: FHREC/2020/01/40/04-05-20) and sought permission from the management of each slaughterhouse before the study commenced. All study participants signed the written informed consent form detailing the purpose and benefit of the study before sample collection. The study participants were also assured of confidentiality of the information obtained. All procedures were performed in accordance with the ethics committee’s guidelines and requirements.

## Supplementary Information

Supplementary Information 1.

Supplementary Information 2.

## Data Availability

All data generated or analyzed during this study are included in this published article [and its supplementary information files].
