# Supplementary material for: Prevalence and risk factors for faecal carriage of multidrug resistant Escherichia coli among slaughterhouse workers
Source: Sci Rep. 2021 Jun 25;11:13362. doi: 10.1038/s41598-021-92819-3 (PMC8233315; doi:10.1038/s41598-021-92819-3)
Supplement: Supplementary file 2 — Supplementary Information 2. [file 41598_2021_92819_MOESM2_ESM.docx]

# QUESTIONNAIRE

**Prevalence and risk factors for faecal carriage of multidrug resistant *Escherichia coli* among slaughterhouse workers**

**Introduction and consent section:**

**INFORMED CONSENT**

Greetings. My name is **Mabel Aworh-Ajumobi** and I am a **Fleming fund fellow** with the Department of Veterinary and Pest Control Services, Federal Ministry of Agriculture & Rural Development, Abuja.

My team is conducting a study that asks women and men questions about working in the abattoir. We would also be collecting your stool sample that will be tested for the presence of *E. coli* organism. This organism is sometimes present in the animals, which are slaughtered at the abattoir and can infect the abattoir worker making him or her sick. You will be told the result of this test later.

This study will take place between June and October, 2020. We would very much appreciate your participation in this study. This information will help the government to plan better health care services for the people of Nigeria. Whatever information you provide will be kept strictly confidential. Participation in this study is voluntary, and if we should come to any question you don't want to answer, just let us know and we will go on to the next question. However, we hope that you will participate in this study since your views are important.

Would you like to participate in this study? **Yes**  **No**

Signature of respondent: ______________________

Date:_______________

| **Prevalence and risk factors for faecal carriage of multidrug resistant *Escherichia coli* among slaughterhouse workers** |
| --- |

**Interviewer’s Name: ------------------------------ Date of Interview: ----------**

| **Name of Abattoir: L.G.A: GPS:** |
| --- |
| **SECTION 1: Demographic Data** (Please Tick appropriate Option) |

1. **Name**:---------------------------------------------------------- (Optional) **Phone No**:----------------------- (optional)
2. **Age group**:  18 -25years  26 – 33 years  34 – 41years  > 41 years
3. **Sex**:  M / F (If male skip to **question 5)**
4. If female, are you pregnant? **Yes**  **No**
5. **Marital Status**:  Single /  Married  Divorced  Separated  Widowed
6. **Occupation:** Butcher  Meat seller  livestock trader  Vet/para-vets  Abattoir Cleaners  Others please specify----------------------------------
7. **Educational Level:** None,  Primary,  Secondary,  Tertiary,  Arabic school?  Others
8. **How long have you been working at abattoir?**

**<** 1 year , 1-5 years , 6-10 years , 11- 20 years , > 20 years

1. **Ethnicity:** a)  Yoruba b)  Ibo, c)  Hausa, d)  Fulani e)  Others, (please specify)--------------------
2. **Residence (where do you stay):**  Urban / Rural
3. **Religion**  Christianity  Islam  Others (pls specify)---------------------------
4. **What are your sources of medical information? (Tick all responses that applies)**

Radio  TV Handbills Social media  Hospital/Health care workers  Friends and family  Electronic news  Others (Pls specify) ---------------------------------------

**SECTION 2: Medical History/History of antibiotics Use**

1. Have you had diarrhea (watery stool) within the last 3 months? **Yes**  **No**
2. If No, when was the last time you had diarrhoea? _______________________
3. How often do you have diarrhea?  Weekly, Monthly, Others (please specify)___________
4. When you are ill, what do you usually do? a)  Go to Hospital/See a doctor **b)**  Go to the Chemist c)  Buy medicine from drug hawkers? d)  Buy local herbs e)  Call a friend/ family member f)  Consult a traditional healer g)  Decide for myself which medicine to take h)  Ask the internet i)  Others please specify -------------------
5. If response to 4 is **g**, then how many times have you decided for yourself in the past 3 months?
6. Once 2. Twice 3. Thrice 4. More than 3 times 5. None
7. If response to 4 is **a** or **b**, then in the past 3 months, on what basis did you take your medicines to treat your health problems?
8. Based on physician’s advice a) Yes b) No
9. Based on past experience with similar illness a) Yes b) No
10. Based on advice from relatives and friends a) Yes b) No
11. Based on information from media a) Yes b) No
12. Have you taken any antibiotics within the last month? **Yes**  **No**

If yes, since when?______________________

If No when was the last time you took antibiotics?______________________

1. What is the name of the antibiotics you take commonly?  Flagyl  Tetracycline  Septrin   cannot remember  others pls specify ------------------------------------------
2. What is the name of the last drug (antimicrobials) you took in the last 3 months? -----------------------
3. What illness did you try to treat? ------------------------------------------
4. Who diagnosed the illness?-------------------------------------------------
5. Did the drugs work for you or make you feel better? a) Yes b) No c) Cant remember d) Don’t know
6. If you are asked to take antibiotics, do you normally finish your dosage?  Yes  No
7. Do you stop taking your drugs when you feel better?  Yes  No
8. If you don’t feel better after taking all the medicine, what do you do? -------------------------------------------------------------------------------------------------------------------------------------------------------------
9. When last did you take antibiotics
10. On that occasion, did you get the antibiotics (or a prescription for them) from a health professional (e.g doctor, Nurse, Pharmacists)?  Yes  No  Cant remember
11. On that occasion, did you get advice from a health professional (e.g doctor, Nurse, Pharmacists) on how to take them?  Yes I received advice on how to take them (e.g. with food, for 7 days)

No  Cant remember

1. On that occasion, where did you get the antibiotics?

a)Medical store or pharmacy

1. Stall or hawker
2. 3. The internet
3. 4. Friend or family member
4. 5. I had them saved up from a previous time
5. 6. Somewhere/someone else
6. 7. Can’t remember

| **SECTION 3: Other Exposure Factors** |
| --- |

1. Do you keep animals at home? **Yes**  **No**
2. What type of animals do you keep?  Cattle  Sheep,  Goats,  chickens  Others please specify----------------------------------------------------
3. Do you live in a farm premises where animals are kept? **Yes**  **No**
4. Do you drink fresh milk (madara / nono)? **Yes**  **No**
5. Do you boil fresh milk before drinking? **Yes**  **No**
6. Do you consume any milk products like cheese, yoghurt etc?

**Yes**  **No**

| **SECTION 4: Work Related Exposure Factors** |
| --- |

1. Do you handle animals at work? **Yes**  **No**
2. Do you wash your hands after touching the animals Yes  No
3. Do you wash your hands with soap and clean water after touching animals Yes No
4. Where do you get your animals for slaughter from? _____________________
5. Have you ever handled fetuses, after birth, etc while slaughtering animals? **Yes**  **No**
6. Do you slaughter animals at the abattoir? **Yes**  **No**
7. Do you sell meat? **Yes**  **No**
8. Have you accidentally cut yourself when slaughtering animals or cutting meat? **Yes**  **No**
9. Are you still involved in slaughter/meat processing when you have a cut/wound?**Yes**  **No**
10. Do you process any animal by-product like wool, hides and skin, or organic manure**Yes**  **No**
11. Do you often collect animal waste to be used as manure? **Yes**  **No**
12. Do you wear gloves when working or cleaning out waste at the abattoir? **Yes**  **No**
13. Do you eat while working at the abattoir? **Yes**  **No**
14. Do you eat raw or uncooked meat? **Yes**  **No**
